# Unmasking the enigma: A case of Fumarate Hydratase-deficient renal cell carcinoma

**DOI:** 10.1016/j.ijscr.2023.109054

**Published:** 2023-11-13

**Authors:** Mirza Rameez Samar, Wajiha Khan, Yasmin Abdul Rashid, Azmina Tajuddin Vali Mohammad

**Affiliations:** a Department of Medical Oncology, The Aga Khan University Hospital, Pakistan; b Department of Medicine And Surgery, Dow University of Health Sciences, Karachi, Pakistan

**Keywords:** Fumarate hydratase, Tyrosine kinase inhibitors, Renal cell carcinoma, Metastatic disease, Erlotinib, Bevacizumab

## Abstract

**Introduction:**

Fumarate Hydratase-deficient-renal cell carcinoma (FH-dRCC) is an uncommon and extremely aggressive variant of renal cell carcinoma (RCC) resulting from inactivating mutations in the tumor suppressor gene, fumarate hydratase (FH).

**Case presentation:**

We report a case of a young male who presented with the complaint of painless hematuria. Upon workup, he was found to have a renal mass with bony metastases. The histopathology was consistent with renal cell carcinoma with features of FH-deficient variant. Germline testing showed a pathogenic mutation in the FH gene. He was started on a treatment combination of Pembrolizumab and Axitinib along with Zoledronate for bone metastasis. His response to the combination therapy was short with early progression of disease. He was switched to a second-line treatment Bevacizumab and Erlotinib, which achieved significant disease response.

**Discussion:**

Systemic therapy is the mainstay of treatment for metastatic disease. Although the novel agents approved for other subsets of RCC have been used, the responses are grim. There is no consensus on the sequence of further lines of treatment for FH-dRCC and is based on the physician's choice, availability of the drugs, cost, toxicity, and tolerance of the patient.

**Conclusion:**

This case report emphasizes the occurrence, presentation, management and prognosis of FH-dRCC, which is an aggressive entity, presenting at a young age with early distant metastases, not diagnosed appropriately due to its poorly characterized cytologic features. Being an infrequent neoplasm, it is an area that warrants oncological exploration to improve outcomes in these individuals. The combination of Erlotinib and Bevacizumab provides promising outcomes in terms of progression-free survival.

## Highlights

• Fumarate hydratase-deficient renal cell carcinoma is a hereditary condition resulting from a mutated fumarate hydratase gene.
• It is a highly aggressive tumor and is notorious for presenting in young adulthood with early distant spread.
• This report highlights an unusual metastatic renal neoplasm with poor response to traditional therapy.
• The combination of Erlotinib and Bevacizumab offers a light of hope for this uncommon kidney cancer subset.

## 1 Introduction

Fumarate hydratase-deficient renal cell carcinoma (FH-dRCC) is a highly invasive and unusual subtype of renal cell carcinoma (RCC) that occurs mostly as part of an autosomal dominant syndrome called, hereditary leiomyomatosis renal cell cancer syndrome (HLRCC). It results due to the inactivating mutations in a tumor suppressor gene called fumarate hydratase (FH), found in 1q42.3-q43 locus, characterized by a germ-line mutation of one allele and somatic mutation of the other allele.

FH is an intracellular enzyme that is exclusively involved in the tricarboxylic acid cycle, where it converts fumaric acid to malic acid. The functional defect in the FH gene results in the accumulation of FH and lowering of intracellular iron levels which in turn inhibits hypoxia-inducible factor prolyl hydroxylase and elevates the level of hypoxia-inducible factor (HIF) within the cells. This increase in HIF regulates the transcription of genes involved in cellular growth, angiogenesis, survival, or apoptosis, thus promoting tumorigenesis. (1) Though other mutations such as Frameshift mutations, nonsense mutations, insertion, deletion, or splice site mutations have also been seen, FH-dRCC is most commonly associated with missense mutations. (2)

Though the age of disease onset can vary from 10 to 90 years old, the reported age of onset is 20–25 years. (3) The majority of the time, these tumors go undiagnosed because the cytological characteristics of these tumors have not been carefully studied until last year when FH-dRCC was recognized as a separate entity by the World Health Organization (WHO) in 2022. (4) As FH-dRCC is rarely reported, the exact incidence is not known. Although novel therapy for advanced RCC has been extrapolated with minimal responses. To date, few case studies or reviews have shed light on this distinct category of RCC as well as the role of a combination of Erlotinib, an epidermal growth factor receptor (EGFR) inhibitor, and Bevacizumab, a vascular endothelial growth factor (VEGF) inhibitor.

Here, we report a case of a young adolescent, who was diagnosed with metastatic RCC, harboring FH mutation, with an exceptional response to a combination of Erlotinib and Bevacizumab in a second-line setting.

The work has been reported in line with the SCARE criteria (5).

## 2 Case presentation

A 22-year-old male, resident of Karachi, and university student, with no known co-morbid illnesses, presented with a short history of self-resolving painless hematuria that lasted for 2 days. The urinary frequency was normal (three to four times per day). There was no associated history of nocturia, urgency, or dysuria. He denied a history of any medical condition or surgical intervention. There was no history of allergies and addiction. Family history was pertinent for renal cancer in his paternal uncle.

**Fig. 1:**
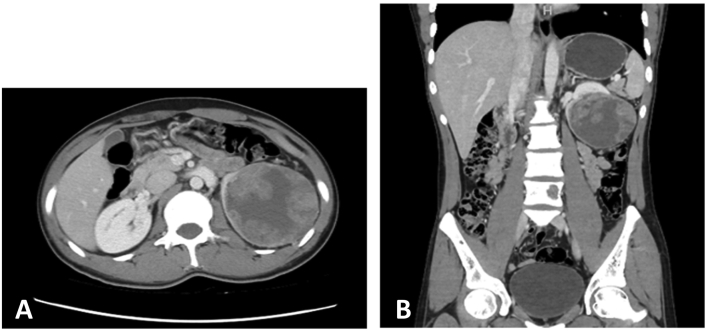
Computed tomography of the abdomen revealing a large heterogeneous left renal mass (A) axial view and (B) coronal view.

His physical and systemic examination was unremarkable. Laboratory investigations including complete blood count, liver function test, and biochemistry were within normal limits. Urinalysis revealed protein+30 with numerous red blood cells and bacteria which prompted further workup with abdominal ultrasound that showed an irregular cystic lesion, with internal echoes and thin septations, at the lower pole of the left kidney, measuring 7.9 × 7.7 cm. This was followed by a contrast-enhanced computed tomography (CT) of the abdomen that highlighted a well-defined rounded lesion, emerging from the lower pole of the left kidney with enhancing cystic components measuring 87 × 82 mm. The lesion was abutting the tail of the pancreas with loss of fat plane superiolaterally, and abutting the left psoas muscle with loss of fat plane posteromedially, and the left lateral abdominal wall laterally. Multiple hypodense lesions were identified in L1-L4 vertebrae, ischium, and roof of left acetabulum suggestive of metastatic deposits. (Fig. 1).

Bone scintigraphy revealed donut-shaped mild tracer uptake involving the L4 vertebrae, correlating with a large osteolytic lesion seen in recent CT. He underwent a left radical nephrectomy. Intra-operatively, a well-circumscribed mass, localized at the lower pole of the left kidney was appreciated, which was not involving the adrenal gland. Histology of the resected lesion showed a high-grade tumor limited to the kidney with prominent tubular, tubulocystic, and tubulopapillary patterns without any sarcomatoid or rhabdoid features. There was no tumor necrosis or lymphovascular invasion. Pathologically, it was staged as pT2aN0 i.e. stage II-A. Immunohistochemistry (IHC) revealed cytokeratin 7 negative, AMACR positive, CD10 positive, and WT-1 negative. Given the gross, histological, and IHC findings, a diagnosis of FH-dRCC was suspected.

Post-operatively, he was started on a combination of intravenous Pembrolizumab, at 200 mg once three weekly, with per-oral Axitinib, at 5 mg twice a day and intravenous Zoledronic acid, every three monthly. Meanwhile, in view of his early age at diagnosis and positive family history, a genetic test was performed which came out to be a pathogenic variant in FH [c.698G>T (p.Arg233Leu)].

**Fig. 2:**
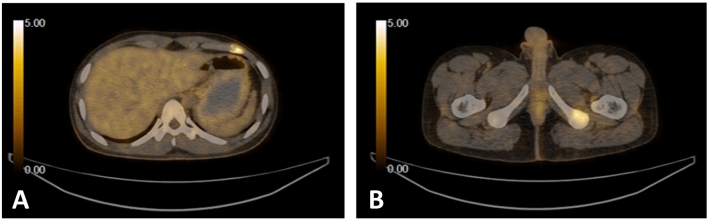
PET-CT demonstrating FDG avidity (A) over the left 6th rib and (B) left ischium.

After completion of eight cycles of the above regimen, positron emission tomography-computed tomography (PET-CT) of the whole body was carried out, which demonstrated osteolytic metastases with interval regression in fluorodeoxyglucose (FDG) avidity over left ischium, acetabulum, L1 and L4 vertebrae; significant interval progression in FDG avidity over the left 6th rib anteriorly; and interval appearance of hypermetabolic marrow deposits involving the left scapular bone, the right 7th rib, T5, and T11 vertebrae. (Fig. 2).

**Fig. 3:**
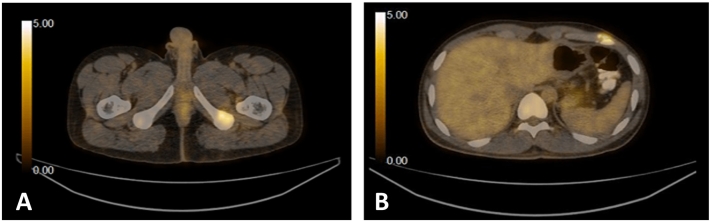
PET-CT demonstrating (A) stable FDG avidity over the left 6th rib and (B) improvement in FDG uptake over the left ischium.

Keeping in view this metabolically progressive disease and after a thorough review of the literature, the patient was switched to a combination of intravenous Bevacizumab, at 10 mg/kg given three weekly and per-oral Erlotinib at 150 mg once daily. After completing 6 cycles of the above regimen, PET-CT was repeated which demonstrated mild FDG avid focus over left Gerota's fascia inferiorly without any significant interval change and demonstration of FDG avid osteolytic metastasis with significant interval reduction in metabolic activity. (Fig. 3) On account of the stable disease process, he is currently advised to continue the same regimen and is scheduled to get a repeat PET-CT in three months.

## 3 Discussion

FH-dRCC is a high-grade carcinoma, divided into a predominant hereditary form called HLRCC and a less prevalent sporadic form. (6) This gross, solitary, and unilateral tumor has histologically been characterized by various patterns such as tubular, tubulocystic, solid, microcystic, or papillary. It has eosinophilic cytoplasm and high-grade nuclei. (7) FH-RCC is an aggressive counterpart, that has the attributes of early age of onset, atypical imaging manifestations, variable pathological patterns, difficult clinical diagnosis, and poor response to traditional drug treatment. (8) High-grade FH-dRCCs are associated with progression and/or death from disease in 30–80 % of cases. (6)

The common presentation involves hematuria and urinary tract irritation with pain either in the loin or back region. Similarly, our patient also presented with an acute history of hematuria without any urinary irritation or pain. Histological and immunochemical staining, as well as analyses of tissue samples, are the most crucial tests to diagnose this clinically relevant subtype, especially for tailoring a treatment strategy. In a study by Trpkov et al., 93 % of FH-dRCCs showed two or more developmental patterns and had at least focal macro-nucleoli. Among the different patterns described above, papillary was the most frequent (74 %) and dominating (59 %) pattern, whereas solid (44 %), cribriform (41 %), tubulocystic (41 %), and cystic (33 %) were also frequent patterns in declining order. (9) This was also seen in our case which demonstrated prominent tubular, tubulocystic, and tubulopapillary patterns.

The management of metastatic RCC involves systemic therapy with palliative intent. On account of the lack of data in this patient subgroup, treatment options are often extrapolated from the studies involving other subtypes of RCC. Over the past decade, several new therapies, either alone or in combination, have been approved, including mammalian target of rapamycin (mTOR) inhibitors such as Everolimus, multi-targeted tyrosine kinase inhibitors (TKIs) namely Sunitinib, Pazopanib, Cabozantinib, and Lenvatinib; and the immune checkpoint inhibitors (ICI) including Pembrolizumab, Nivolumab and VEGF/ICI combination therapies. Of these, mTOR inhibition alone had no response, whereas dual inhibition of mTOR as well as VEGF pathways, resulted in an objective response rate (ORR) of up to 44 %. (10,11) Even the use of TKIs as single agents, demonstrated an ORR of up to 64 % with a time to advancement of 11.6 months, outperforming mTOR inhibitors. (11)

Apart from the standard targets, EGFR activation can also cause HIF stability by activating the phosphoinositide 3-kinase (PI3K)/AKT pathway, which in turn, promotes tumorigenesis whereas inhibition of EGFR reduces the expression of the HIF target VEGF. This action has given rise to the idea of combining EGFR with VEGF receptor inhibitors. ([12], [13], [14]) In a single phase II study, the combination of Bevacizumab and Erlotinib resulted in an ORR of 72.1 % and a median progression-free survival (PFS) of 21.1 months. (15) Moreover, FH-dRCC patients treated with Bevacizumab plus Erlotinib demonstrated an ORR of 50 % with a median PFS of 13.3 months and a disease control rate of 90 %, in the first-line setting. (16) In another case series, the combination of Bevacizumab and Erlotinib resulted in a PFS of 14 months. (17) Our patient too was initially started on a traditional combination of ICI and TKI with disease progression in 6 months following which he was switched to a combination of Erlotinib plus Bevacizumab which resulted in significant disease improvement.

## 4 Conclusion

FH-dRCC warrants special attention, due to its rare occurrence and highly invasive nature. As the efficacy of routine agents has not been established and considering the response in our case was consistent with the responses highlighted in prior studies, it can be hypothesized that the combined use of Erlotinib and Bevacizumab could be of significant benefit in FH-dRCC, however, larger trials are needed to validate the efficacy of this treatment regimen.

## Abbreviations

FH-dRCC: Fumarate hydratase-deficient renal cell carcinoma
RCC: Renal cell carcinoma
HLRCC: Hereditary leiomyomatosis renal cell cancer syndrome
FH: Fumarate hydratase
HIF: Hypoxia-inducible factor
WHO: World Health Organization
EGFR: Epidermal growth factor receptor
VEGF: Vascular endothelial growth factor
CT: Computed tomography
IHC: Immunohistochemistry
PET-CT: Positron emission tomography-computed tomography
FDG: Fluorodeoxyglucose
mTOR: Mammalian target of rapamycin
TKI: tyrosine kinase inhibitors
ICI: Immune checkpoint inhibitors
ORR: Objective response rate
PI3K: Phosphoinositide 3-kinase
PFS: Progression-free survival

## Ethical approval

Not applicable as case reports are exempted from the provision of ethical approval in our institute.

Ethical approval was waived for this study as it was a case report and The Aga Khan University Hospital's Ethical Review Board exempts case reports for Ethical Approval Certificate.

## Funding

Not applicable.

## Credit authorship contribution statement

Mirza Rameez Samar conceptualized and designed the study, drafted the initial manuscript, and finalized the manuscript after editing.

Wajiha Khan drafted the initial manuscript.

Yasmin Abdul Rashid edited the manuscript and looked over the submission process.

Azmina Tajuddin Vali Mohammad critically reviewed the manuscript for important intellectual content.

## Guarantor

Mirza Rameez Samar.

## Registration of research studies

Not applicable.

## Consent to participate

Written informed consent was obtained from the patient for publication of this case report and accompanying images. A copy of the written consent is available for review by the Editor-in-Chief of this journal on request.

## Provenance and peer review

Not commissioned, externally peer-reviewed.

## Declaration of competing interest

The authors declare that they do not have any competing interests.

## Data Availability

The data for the current study is available from the corresponding author at reasonable request.
